# Coupling template nanocasting and self-activation for fabrication of nanoporous carbon

**DOI:** 10.1038/srep38176

**Published:** 2016-11-30

**Authors:** Lingjun Kong, Mingxiang Liu, Zenghui Diao, Diyun Chen, Xiangyang Chang, Ya Xiong

**Affiliations:** 1 School of Environmental Science and Engineering, Guangzhou University, Guangzhou, 510275, P. R. China; 2 Guangdong Provincial Key Laboratory of radioactive contamination control and resources, Guangzhou, 510275, P. R. China; 3 Guangdong Provincial Key Laboratory of Environmental Pollution Control and Remediation Technology, Sun Yat-sen University, Guangzhou, 510275, P. R. China; 4 South China Sea Institute of Oceanology, Chinese Academy of Sciences, Guangzhou 510301, China

## Abstract

Hierarchical nanoporous carbon (NPC) with great surface area and developed pore size distribution has been intently concerned. Herein, we report a facile method coupling template nanocasting and self-activation to fabricate nanoporous carbon with continuous micro, meso and macro pores, in which CaCO_3_ acted as template and activation reagent while the flour was the carbon precursor. Effects of mass ratio of CaCO_3_ to flour and carbonized temperature on the pore structures of NPC were investigated by nitrogen adsorption-desorption isotherms and SEM analysis. Another kind of carbon was prepared by directly mixed powder CaCO_3_ with flour carbonized at 800 °C (NPC-p) to comparatively investigate the pore fabricating mechanism. Results shown that carbonized at 800 °C was favorable to fabricate the continuous macro, meso and micro pores. The resulted NPC in a mass ratio of 1 to 2 had the considerable S_BET_ and V_T_ of 575.4 m^2^/g and 0.704 cm^3^/g, respectively. Only surface activation was observed for NPC-p. Nanocasting of the powder CaCO_3_ contributed to fabricate macropores and the CO_2_ activation contributed to meso- and micropores. Coupling activation and nanocasting effect due to the decomposition of CaCO_3_ template into CO_2_ and CaO was ascribed to synthesize the nanoporous carbon.

Hierarchical nanoporous carbon with great surface area and developed pore size distribution has been intently concerned due to its widely application in adsorption, catalysis, energy storage and CO_2_ capture^1^. Because the hierarchical pores have exceptional surface area and facilitation in the transport of the large molecules through the bulk, overcoming the size restriction. For example, our previous study synthesized sludge derived char with hierarchical “pore in pore” structure by combining the citric acid as soft template and zinc chloride as activation reagent, enhancing its adsorption ability to toluene^2^. Li *et al*. reviewed that the hierarchical pores performed the advantageous opportunities in energy storage and conversion^3^. Liu *et al*. synthesized hierarchical mesoporous/microporous carbon, favoring the electrolyte penetration and transformation, the micropores drilled on the mesopore-walls resulted in the increase in the specific surface area to provide more sites for charge storage^4^. Order hierarchical mesoporous/macroporus carbon catalyst not only facilitates electrolyte immersion and diffusion but also has effective space for O_2_ diffusion and conversion^5^. Consequently, synthesis of the hierarchically porous carbon will be widely concerned in the future.

Varied methods including activation^6^,^7^,^8^,^9^,^10^, carbonization of polymer^11^,^12^,^13^,^14^, nanocasting^15^,^16^,^17^,^18^,^19^ with template has been adopted to synthesize these carbon materials. Activation processes could be classified into physical and chemical activation according to the evolved activation reagents. In which, the physical activation is related to CO_2_^20^ and steam^21^ carbonized at about 800 °C while chemical activation need H_3_PO_4_^22^,^23^, NaOH^24^, KOH^25^, ZnCl_2_^26^ *et al*. Equipment corrosion is occurred and activation reagent is expansive for chemical activation^27^, which had been the bottleneck for their commercialization, hampering its widespread application. Xu *et al*. had prepared porous carbon with polyvinylidene chloride precursor and adjusted the textural properties by varying the carbonization temperature between 400 and 900 °C^28^. After that, polyvinylidene fluoride derived hierarchically porous carbon was widely paid more interesting^29^,^30^. The cost is determined by the polyvinylidene chloride precursor.

Nanocasting with template, which was pioneered by Knox *et al*. at 1986^31^, is also believed to be an efficient method for preparing porous carbon. In this method, the organic carbon precursors (e.g., sucrose, furfuryl alcohol, phenol resins, polystyrene, mesophase pitches) are infiltrated into the mesoporous solid templates, and further being subjected to be carbonized at oxygen-free atmosphere. The solid templates were removed by acid washing for fabricating the pores. This method is favorable to control the pore size distribution^32^,^33^, and the pore size could be adjusted and designed by choosing the template but limited to the thickness of the walls of the templates.

The zeolites are widely adopted as inorganic templates for controlment of the pore size of porous carbons since the thickness of the walls is ranged from 0.1 to 3 nm^34^. The resulted pore structures were mainly micro- and mesopores. Besides, colloidal templates were usually applied to synthesize meso- and macropore structure. The used colloidal template included polymers, inorganic oxides and metals. For example, the silica sphere in a diameter of 150 to 300 nm was usually adopted to synthesize three-dimensional macropores carbon^35^. Monodisperse silica spheres in a diameter of 30–100 nm were conducted as colloidal templates and sucrose was conducted as carbon precursor to synthesize uniform porous carbon with a diameter ranged from 20–80 nm^34^. Although the meso- and macropores take the advantages in mass transfer being applied in adsorption and catalysis, the meso and macro-porous carbons usually lead to relatively low surface area (S_BET_) with low reactive site because the S_BET_ is dependent on the micro- even narrow micropores, but the narrow pore size is not beneficial to the adsorption and transfer of the pollutants to some degree.

Recently, it has been proposed that a hierarchical porous texture with balanced micropores, mesopores and macropores is beneficial for carbon materials as they can afford both high capacitance and good rate capability^36^. Hierarchical nanoporous carbon with broad pore size distribution is promising considering its application. The synthesis of various porous carbon materials having hierarchical structures is expected in future. Whereas, narrow and micro-pores are mainly formed during the activation^37^, combining the pores fabricating effect of activation and nanocasting for coupling fabricating macro, meso and micropores should be greatly promising.

The CaCO_3_ nanoparticles, as a hard template, are commercially available and environmental friendly, and can be easily removed using HCl aqueous solution instead of corrosive HF, making the nano- CaCO_3_ template method much simpler, easier to operate and more cost-efficient than the conventional silica template method^38^. More important, the CaCO_3_ could be decomposed into CO_2_, which is a kind of physical activation reagent^39^. However, the detailed contribution and coupling mechanism of nano CaCO_3_ to fabrication of micro-, meso- and macropore were not reported. Herein, we report coupling template nanocasting and self-activation method to fabricate nanoporous with continuous micro, meso and macro pores, in which the micro powder calcium carbonate (CaCO_3_) lower than 12500 meshes was adopted as template and activation reagent while the easily available flour was adopted as the carbon precursor. Varied mass ratio of CaCO_3_ and flour were impregnated to form gels and carbonized at high temperature, further being washed by acid solution to control its pore size. The pore structures were characterized by nitrogen adsorption-desorption isotherms and SEM analysis to investigate the pore fabricating mechanism.

## Results and Discussion

### Pore-fabrication in the presence of CaCO_3_

**Figure 1:**
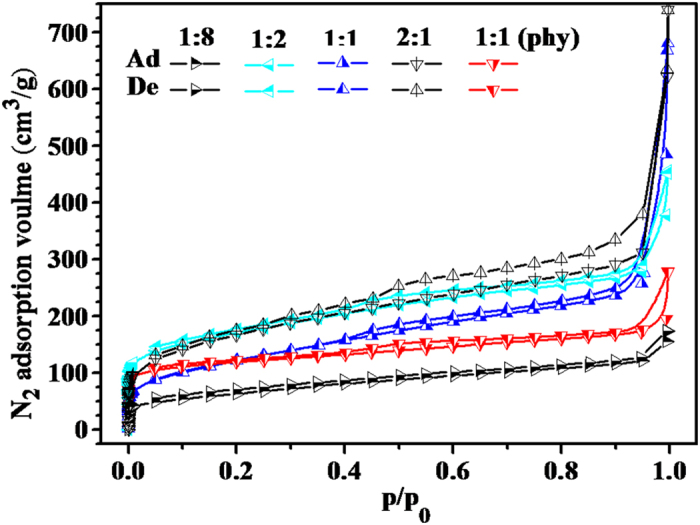
Effect of CaCO_3_ addition content on the Nitrogen adsorption-desorption curves of NPC carbonized at 800 °C.

Without addition of CaCO_3_ powder, the resulted carbon carbonized at 800^ ^°C had poor S_BET_ and total pore volume, which was only 25.211 m^2^/g and 0.0265 cm^3^/g, respectively. In order to further investigate the effect of CaCO_3_ template on enhancing the fabrication of pores, NPCs were prepared by impregnation of CaCO_3_ to flour in varied mass ratio carbonized at 800 °C. The resulted NPCs were labeled as NPC-i-1, NPC-i-2, NPC-i-3 and NPC-i-4 according to the mass ratio of CaCO_3_ to flour of 1:8, 1:2, 1:1 and 2:1, respectively. The nitrogen adsorption-desorption isotherms of them were presented in Fig. 1. The adsorption isotherms of NPC-i-1, NPC-i-2, NPC-i-3 and NPC-i-4 were all belong to Type II according to IUPAC classification^40^. Monolayer adsorptions at low relative pressure were observed for the all NPCs with varied mass ratio, indicating the occurrence of rich micropores by CO_2_ activation. When the relative pressure increased to about 0.995, the nitrogen adsorption amounts of them were all increased greatly. But the detailed results were different corresponding to the varied mass ratio of CaCO_3_ to flour. Obviously, the increase in the adsorption amount was the highest for NPC-i-4 while it was the lowest for NPC-i-1. In a word, the adsorption amounts of them at relative pressure of 0.995 followed the order: NPC-i-1< NPC-i-2< NPC-i-3< NPC-i-4. The adsorption amount at relative pressure about 0.995 was due to the nitrogen filling in the pores. As shown in Table 1, the total pore volume (V_T_) for NPC-i-1, NPC-i-2, NPC-i-3 and NPC-i-4 was 0.269, 0.704, 1.177 and 1.144 cm^3^/g, respectively, being far higher than the carbon without CaCO_3_. Clearly, the total pore volumes are partly consistent with the added CaCO_3_ content for NPC-i-1, NPC-i-2 and NPC-i-3. It means that the addition of CaCO_3_ is favorable for fabricating the macropores. More important, it quite depends on the amount of CaCO_3_.

**Figure 2:**
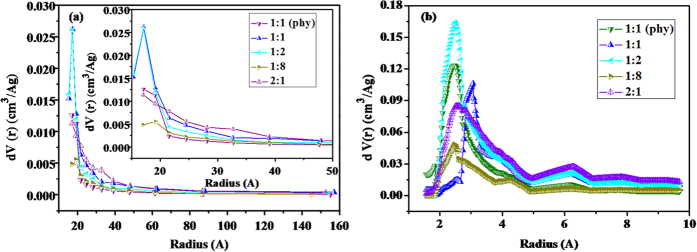
Effect of CaCO_3_ addition content on the pore size distribution of NPC carbonized at 800 °C (**a**) BJH method, (**b**) HK method.

Moreover, the pore size distribution calculated by BJH method shown in Fig. 2(a) was different. The pore radius for NPC-i-1 and NPC-i-2 was mainly lower than 25 A. While considering the NPC-i-3 and NPC-i-4, their radius were not only located at range of 10 to 25 A, but also located at higher than 25 A. The mesopore volume shown in Table 1 increased as the increase in the CaCO_3_ content for NPC-i-1, NPC-i-2 and NPC-i-3, indicating the addition of CaCO_3_ also favors to fabricate mesopores as similar to macropores. The pore size was related to the CaCO_3_ content. This result could be regarded as the template nanocasting effect since the powder CaCO_3_ was regarded as efficient template for fabricating the pores^38^.

However, as shown in Fig. 2 and Table 1, some porous characteristics such as pore size distribution and S_BET_ of the test nanoporous carbon were different considering the mass ratio of CaCO_3_ to flour. The pore radius of NPCs was mainly located from 2 to 5 A. First, the increase in the CaCO_3_ content could favor the fabrication of the micropores. The NPC-i-2 had higher micropore distribution than NPC-i-1 in the range of 2 to 5 A, the micropore volume and S_BET_ of NPC-i-1 were only 0.09 cm^3^/g and 225.1 m^2^/g, respectively, they are the lowest among the tested NPCs. Considering the micropore volume and S_BET_ of NPC-i-2, they increased to 0.250 cm^3^/g and 575.4 m^2^/g, confirming the pore fabrication effect in the presence of CaCO_3_ template. It is because that the CaCO_3_ micro powder as template impregnated in the NPC was decomposed into CaO and CO_2_ during carbonization at 800 °C^39^. While it is well known that CO_2_ is a kind of efficient activation for fabricating pores, thus, the formed carbon enwrapped around the micro powder CaCO_3_ template could be activated by the decomposed CO_2_, fabricating the micropores that enlarge the micro volume and S_BET_. But on the other hand, the further increase in the CaCO_3_ content did not result in the increase in the micropore volume and the S_BET_ but leaded to decrease in some degree, suggesting that the addition of CaCO_3_ as template could fabricate the pores of NPCs, but a suitable CaCO_3_ content is determined for fabricating the micropores especially for the NPC-i-2 with the highest micropore volume when the mass ratio of CaCO_3_ to flour is 1:2.

Once the CaCO_3_ content increased to some degree, one hand, the CaCO_3_ template played nanocasting effect on fabricating the meso and macropores, another hand, the CaCO_3_ played activation role in fabricating micropores, leading to the increase in the micropore volume, but excessive activation could enlarge the pores, resulting in the increase in the total pore volume and the decrease in the micropore volume. These facts could directly proved by the facts of the decrease in the S_BET_ and the increase in the total pore volume as the increase in the mass ratio of CaCO_3_ to flour shown in Table 1. In a word, the pore fabricating effect by CaCO_3_ as template could be contributed to the coupled nanocasting and activation effect, resulting in a broad and continuous pore size distribution, but excessive CaCO_3_ could lead to the enlargement in the pores.

### Nanoporous carbon carbonized at varied temperature

**Figure 3:**
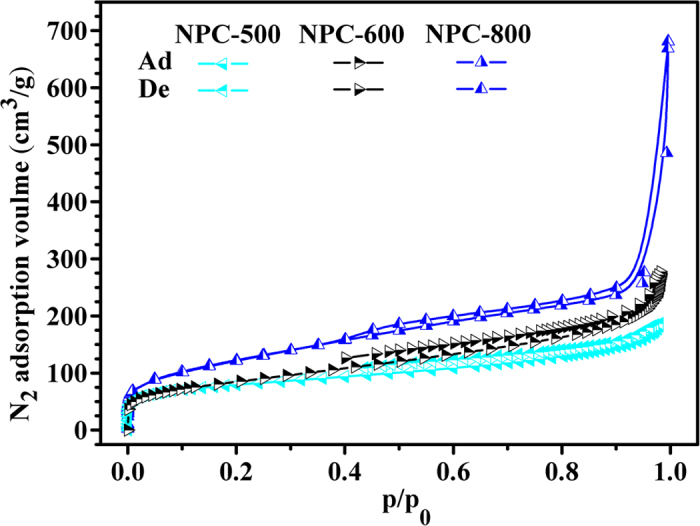
Nitrogen adsorption-desorption isotherms of NPCs carbonized at different temperature (Ad: adsorption, De: desorption, Flour: CaCO_3_ = 1:1).

The NPCs were prepared at varied temperature to explore the pores fabricating process. The resulted NPCs were named as NPC-500, NPC-600 and NPC-800, in which the number presented the carbonized temperature. Nitrogen adsorption and desorption isotherms of NPC-500, NPC-600 and NPC-800 were presented in Fig. 3. The isotherms are belonged to Type II according to IUPAC classification^40^. They all can be ascribed to the macroporous materials. At low relative pressure, the adsorption of nitrogen mainly occurred by monolayer adsorption on micropores. However, the adsorption amounts keep increasing as the increase in the relative pressure, indicating the multiplayer adsorption. More interestingly, the adsorption amounts keep increasing at the relative pressure of about 0.900 to 0.995, what is ascribed to the adsorption of nitrogen on the macropores. Comparing these adsorption isotherms, clearly, the carbonized temperature significantly affects the adsorption and desorption isotherms, the NPC-800 not only has the highest adsorption amount at low relative pressure, but also has the highest adsorption amount at relative pressure of about 0.995. Especially, the adsorption amount for NPC-800 increased more sharply comparing to the NPC-500 and NPC-600 when the relative pressure increased from 0.900 to 0.995. The results may be due to the highest adsorption amounts of nitrogen on the macropores of NPC-800, indicating the highest macropore volume for NPC-800 as shown in Table 2. In addition, the samples show capillary condensation step at relative pressure about 0.4, indicating the presence of the mesopores. These adsorption isotherms for NPC-500, NPC-600 and NPC-800 comprehensively indicate that adsorption of nitrogen on the micro, meso and macroporoes are all occurred but it is the highest for the NPC-800.

**Figure 4:**
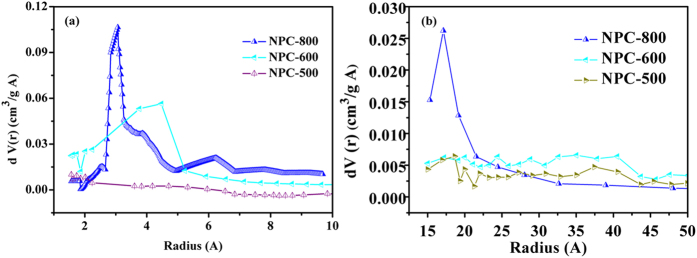
Pore size distribution of NPCs carbonized at different temperature with CaCO_3_ in a mass ratio of 1:1 calculated by (**a**) micropores calculated by HK method, (**b**) macropores calculated BJH method.

Pore size distributions of NPC-500, NPC-600 and NPC-800 were presented in Fig. 4(a) and (b) to discuss the effect of carbonized temperature on the porous structures detailedly. Almost no micropore size distribution peak was found for NPC-500. Once the carbonized temperature increased to 600 °C, the micropore distribution peaks were observed. They are mainly located at r < 5 A. Once the carbonized temperature increased to 800 °C, the volume of micropores located at about radius of 2–5 A and larger than 5 A were greater than that of NPC-600. The NPC-800 had the greatest micro volume of 0.175 cm^3^/g as shown in Table 2. These results could be confirmed by the highest monolayer adsorption amounts onto micropores for NPC-800 at relative pressure. Since the S_BET_ is largely dependent on the volume of the micropores, thus, the NPC-800 had the greatest S_BET_ of 441.2 m^2^/g as shown in Table 2. The S_BET_ of the test NPCs followed the order: NPC-500< NPC-600< NPC800, being agreement with their micropore volume. The results suggested that carbonized at 800 °C with CaCO_3_ template is favorable for fabricating the micropores with radius smaller than 10 A and enlarging the S_BET_.

Moreover, the pore size distribution calculated by BJH method shown in Fig. 4(b) indicates that the pore size distributions of NPC-500 and NPC-600 with pore radius ranged from about 10 to 50 A were similar. But for the NPC-800, its pore size distribution was quite different, especially great pore size distribution peak at the pore radius ranged from 15 to 25 A was observed, indicating the fabrication of mesopores for NPC-800.

**Figure 5:**
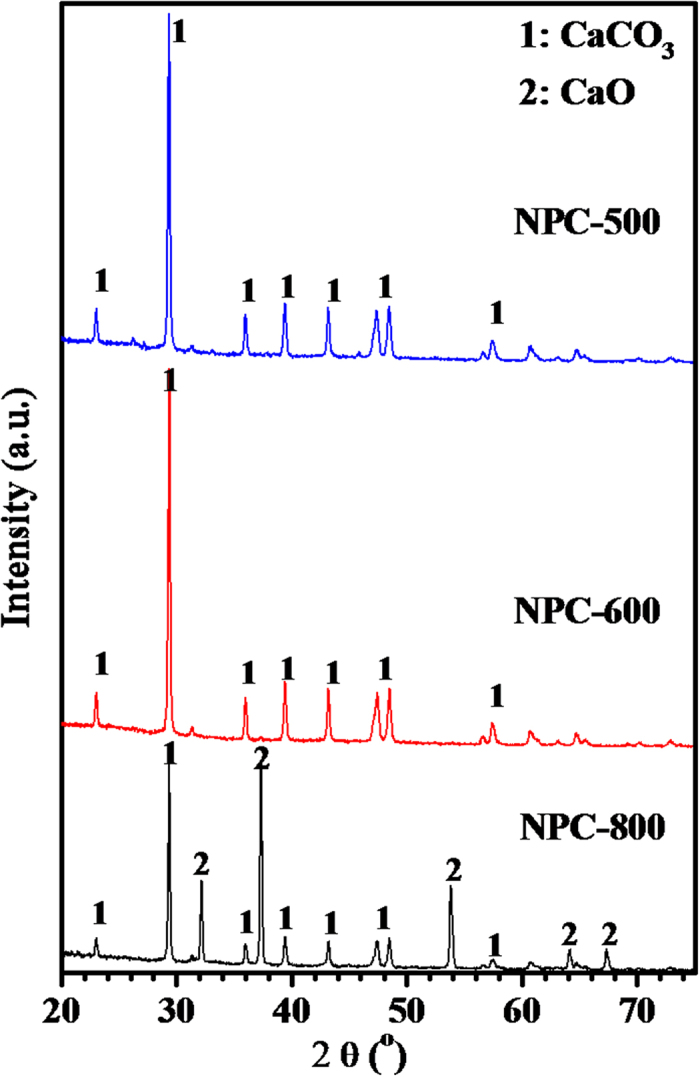
XRD patterns of the NPCs carbonized at varied temperature impregnated with CaCO_3_ powder.

Obviously, among the NPC-500, NPC-600 and NPC-800, NPC-800 had the highest S_BET_ and the richest pore structure. These results indicate that carbonization at 800 °C with CaCO_3_ template is favorable to fabricate the nanoporous. As shown by Fig. 5, the XRD patterns at about 2θ = 32.28, 37.44, 53.92, 64.24 and 67.41°, being ascribed to the CaO, were appeared after being carbonized at 800 °C since CaCO_3_ micro powder as template impregnated in the NPCs was decomposed into CaO and CO_2_ during carbonization at 800 °C^39^. The decomposed CO_2_ is well known for activation, thus leading to the development of micropores. In addition, the impregnated CaCO_3_ template after being decomposed into CaO could occupy the inner space, the decomposition of CaCO_3_ and further removal of the decomposed product of CaO could lead to formation meso and macro pores. This pores fabricating process was regarded as the template nanocasting effect. The NPC-800 had a total pore volume of 1.177 cm^3^/g, it is the highest among the tested NPCs. While considering for NPC-500 and NPC-600, the carbonized temperature is not high enough to make the decomposition of CaCO_3_, no CaO was observed from Fig. 5, the activation of CO_2_ could be ignored, fewer micropores were observed compared to NPC-800, only CaCO_3_ template nanocasting effect would be ascribed to the fabrication of the limited meso and macro pores. Thus, the discrepancies of the poorer porous structure of NPC-500 and NPC-600 than NPC-800 are ascribed to the ignored CO_2_ activation for NPC-500 and NPC-600. The micro powder CaCO_3_ as template takes both nanocasting and activation effect on fabricating rich pores.

### Pore fabricating mechanism

**Figure 6:**
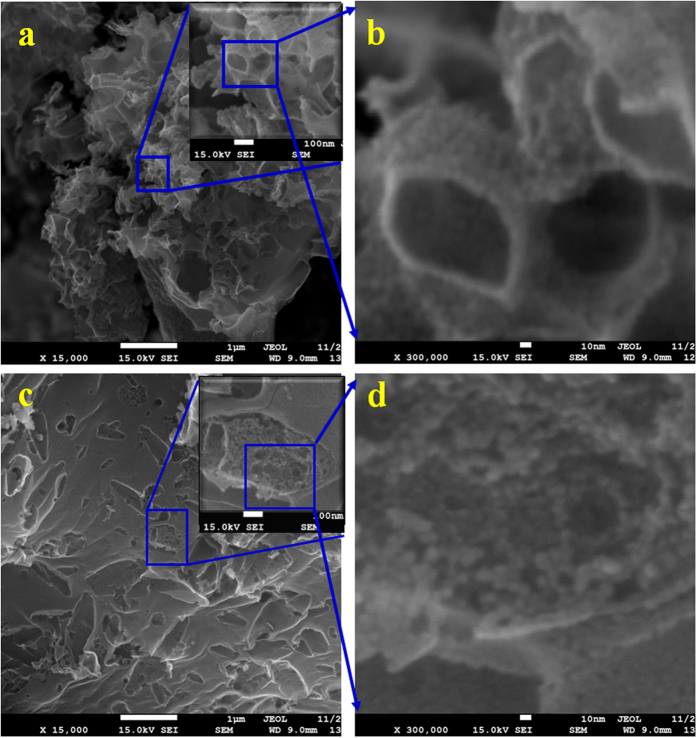
SEM images of NPC-i-3 magnified at (**a**) 15,000, (**b**) 300,000 and NCP-p (**c**) 15,000, (**d**) 300,000.

SEM morphologies of NPCs (Fig. 6) in mass ratio of 1:1 prepared by impregnation and physical mixing were conducted to directly confirm the pore-fabricating mechanism. The Fig. 6(a) and (b) were the NPC-i-3 prepared by impregnation method while the Fig. 6(c) and (d) were the carbon (NPC-p) prepared at 800 °C by directly mixing of powder CaCO_3_ and flour in a mass ratio of 1:1 without impregnation. A large amount of pores were observed for NPC-i-3. Obviously, the pore sizes are quite diversified. As seen from the Fig. 6(a) and (b), the pores with diameter ranged from 100–500 nm were observed. Considering the SEM images of the NPC-p (in Fig. 6(c) and (d)), almost no macropores in diameter of 100–500 nm was observed on the external surface of NPC-p. The V_T_ of NPC-p was far lower than NPC-i-3 (Table 1). Comparing to the tested samples, nanocasting effect of CaCO_3_ template was not occurred for NPC-p without impregnation, clearly suggesting that only impregnation of CaCO_3_ contribute to nanocasting effect on fabricating the macropores.

More interestingly, as shown in Fig. 6, numerous pores with diameter smaller than 10 nm were observed on the external and inner surface of the above mentioned macropores. The occurrence of the pores smaller than 10 nm could be proved by the micropores and mesopores distribution as shown in Fig. 2. Meanwhile, numerous pores smaller than 10 nm are also observed on the surface of the NPC-p. These pores in a diameter smaller than 10 nm were fabricated by CO_2_ activation on the external surface.

Comparing to the nitrogen adsorption isotherm and pore size distribution of NPC-p and NPC-i-3with the same mass ratio of CaCO_3_ to flour in Fig. 1 and Fig. 2, we can clearly see that the adsorption isotherm of NPC-p was belong to Type I, meaning the mainly occurrence of micropores. Then the pore size distribution shown in Fig. 2 suggested that the pore size of NPC-p distributed at radius of 2 to 5 A and 10 to 20 A, mainly ascribed to the micro and narrow mesopores. The adsorption amount to nitrogen is obviously lower than that of NPC-i-3. The NPC-p had lower S_BET_, total volume, micro and mesopore volume than NPC-i-3 although the CaCO_3_ content is the same (Table 1). This can be explained by the SEM results that numerous pores with diameter smaller than 10 nm were observed on the both external and inner surface for NPC-i-3. The formed macropores provided more inner and external space for fabricating the micropores, indicating that the CaCO_3_ played preferable effect on fabricating the pores of NPC-i-3 with impregnation method.

Since the CO_2_ activation is favorable in fabricating the micro and mesopores^20^ while the CaCO_3_ was a template for fabricating the mesopores and macropores, no macropores was fabricated and pores smaller than 10 nm were only fabricated on the external surface for NPC-p due to without wrapping of the CaCO_3_ powder. The pore fabrication process of the NPCs-i could be regarded as coupling effect of template nanocasting and activation.

**Figure 7:**
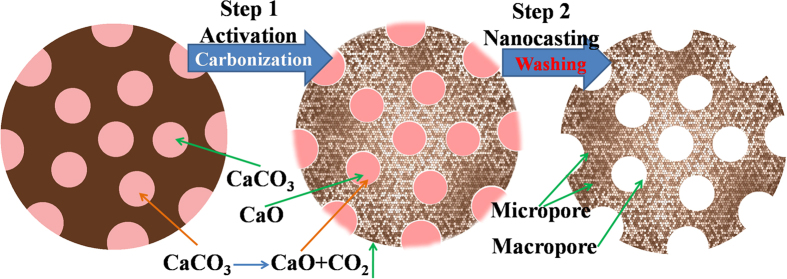
The diagram of coupling nanocasting and activation effect on fabrication of nanoporous carbon.

As shown in Fig. 7, the micro CaCO_3_ particles were impregnated into the gel of the flour, occupying the inner space. After being carbonized, the flour was transformed into carbon. As the temperature increased to 800 °C, the flour was further carbonized, meanwhile, the CaCO_3_ was decomposed into CaO and CO_2_. The formed CO_2_ could be directly reacted with the around carbon to make the around carbon be corroded, meaning the activation for fabricating meso and micropores. Further, the decomposed CaO particles remain in the inner of the carbon was removed by acid washing, leaving the inner pores and forming the macropores. This process could be regarded as the template nanocasting. Finally, macropores bestrewed with numerous micro and mesopores were fabricated by coupled template nanocasting and activation method.

## Conclusion

Impregnated micro CaCO_3_ powder could be taken as template for coupling nanocasting and activation effect on fabricating the nanoprous carbon with broader pore size. The micro CaCO_3_ was decomposed into CaO and CO_2_ at 800 °C. Thus, the decomposed CaO could contribute to fabricate the macro and meso pores by nanacasting when the CaO was removed by acid washing, while the CO_2_ activation could be ascribed to fabricate the micro and mesopores in the process. The resulted NPC-i-2 had the greatest S_BET_ (575.4 m^2^/g) and richest pore structure, resulting in a hierarchically porous structure that numerous micro and mesopores were located on the inner and external surface of the macropores. Coupling effect of nanocasting and activation was ascribed to the pore fabricating mechanism in the presence of impregnated micro CaCO_3_ as template.

## Materials and Method

### Materials

Flour was purchased from the market of Guangzhou (Industry grade). Micro powder calcium carbonate lower than 12500 meshes was purchased from Aladdin Industrial Corporation, Shanghai, China. The other chemical reagents were all purchased from Damao chemical reagent. Co, Tianjin, China (AR).

### Synthesis of nanoporous carbon

The nanoporous carbons were prepared by template nanocasting assisted with activation method. Micro powder calcium carbonate (CaCO_3_) and flour were adopted as template and carbon precursor, respectively. Firstly, the CaCO_3_ and micro flour were mixed in a determined mass ratio, determined volume deionized water was added to the mixtures, and furthered being treated by ultrasound to make the powders be well dispersed to form homogeneous suspension. The suspend mixtures were dried by ultrasound water bath heater at about 80 °C to form gels. The gels were further dried enough at 105 °C in an oven overnight, being taken to an quartz tube (diameter, 40 mm) of programmable tube furnace, carbonized at 600 °C for 2 h and further being carbonized at 800 °C for 2 h under nitrogen flow (heating rate: 20 °C/min). The carbonized samples were washed with 0.1 M HCl solution and deionized water several times until the conductivity became constant to ascertain the ions were removed adequately. Finally, the washed materials were dried at 105 °C overnight, ground and sieved from 60 to 100 meshes. The obtained samples were named as NPC-i-r, in which the letter “i” indicates the impregnation method and the word “r” represented the mass ration of CaCO_3_ to flour. In order to further investigate the pores fabricating mechanism, the dried gels were carbonized at 500, 600 and 800 °C in a mass ratio of 1:1 and washed by aforementioned acid washing, being named as NPC-500, NPC-600 and NPC-800, respectively. And also, the powder CaCO_3_ and flour directly physical mixed in a mass ratio of 1:1 without impregnation and ultrasound mixing were carbonized and washed by aforementioned acid washing. The obtained sample was named as NPC-p.

### Characterization of nanoporous carbon

The pore characteristics of the nanoporous carbon were investigated by nitrogen adsorption-desorption isotherm at 77 K, using an auto-adsorption system (Auto-sorb-6, Quantachrome). The micropores and mesopores distribution were calculated by HK and BJH method, respectively. The chemical state of CaCO_3_ on the carbons were followed by X-ray Diffraction (XRD) using D/max 2200 vpc Diffratometer (Rigaku Corporation, Japan) with a Cu Kα radiation at 40 kV and 30 mA. Scanning electron microscopy (SEM) images were recorded using a JEOL JSM-6330F-mode Field Emission Scanning Electron Microscope (JED-2300).

## Additional Information

**How to cite this article**: Kong, L. *et al*. Coupling template nanocasting and self-activation for fabrication of nanoporous carbon. *Sci. Rep.* **6**, 38176; doi: 10.1038/srep38176 (2016).

**Publisher's note:** Springer Nature remains neutral with regard to jurisdictional claims in published maps and institutional affiliations.

## Figures and Tables

**Table 1:** The S_BET_ and pore volume characteristics of nanoporous carbon produced with different ratio of CaCO_3_ to flour.

Ratio	S_BET_ (m^2^/g)	V_T_ (cm^3^/g)	V_HK_ (cm^3^/g)	V_BJH_ (cm^3^/g)
1:8	225.1	0.269	0.092	0.164
1:2	575.4	0.704	0.250	0.471
1:1	441.2	1.177	0.175	1.045
2:1	327.8	1.144	0.241	0.896
NPC-p	376.7	0.4224	0.1591	0.2614

**Table 2:** The S_BET_ of nanoporous carbon carbonized at varied temperatures.

Temperatures (^o^C)	500	600	800
S_BET_ (m^2^/g)	279.2	309.5	441.2
V_t_ (cm^3^/g)	0.234	0.379	1.177
V_micro_ (cm^3^/g)	0.087	0.114	0.175
